# Does it matter what your reasons are when deciding to disclose (or not disclose) a disability at work? The association of workers’ approach and avoidance goals with perceived positive and negative workplace outcomes

**DOI:** 10.1007/s10926-020-09956-1

**Published:** 2021-02-01

**Authors:** Monique A. M. Gignac, Arif Jetha, Kathleen A. Martin Ginis, Selahadin Ibrahim

**Affiliations:** 1 Institute for Work & Health, 400 University Avenue, Suite 1800, Toronto, ON M5G 1S5 Canada; 2 Dalla Lana School of Public Health, University of Toronto, Toronto, ON Canada; 3 Department of Medicine School of Health and Exercise Sciences, Centre for Chronic Disease Prevention and Management, University of British Columbia, Kelowna, BC Canada

**Keywords:** Disability, Disclosure, Mental health, Episodic disabilities, Work outcomes

## Abstract

Deciding whether to disclose a disability to others at work is complex. Many chronic mental and physical health conditions are associated with episodic disability and include times of relative wellness punctuated by intermittent periods of activity limitations. This research draws on the disclosure processes model to examine approach and avoidance disclosure and non-disclosure goals and their association with perceived positive and negative workplace outcomes. Participants were 896 employed individuals (57.7% women) living with a chronic physical or mental health/cognitive condition. They were recruited from an existing national panel and completed an online, cross-sectional survey. Participants were asked about disclosure decisions, reasons for disclosure/non-disclosure, demographic, work context and perceived positive and negative disclosure decision outcomes (e.g., support, stress, lost opportunities). About half the sample (51.2%) had disclosed a disability to their supervisor. Decisions included both approach and avoidance goals. Approach goals (e.g., desire support, want to build trust, maintain the status quo at work) were significantly associated with perceived positive work outcomes regardless of whether a participant disclosed or did not disclose a disability at work, while avoidance goals (e.g., concerns about losing one’s job, feeling forced to disclose because others notice a problem) were associated with perceived negative work outcomes. The findings highlight benefits and challenges that workers perceive arise when they choose to disclose or not disclose personal health information. By better understanding disclosure decisions, we can inform organizational health privacy and support gaps to help sustain the employment of people living with disabilities.

**Supplementary Information:**

The online version of this article (10.1007/s10926-020-09956-1) contains supplementary material, which is available to authorized users.

## Introduction

The numbers of individuals living with a physical or mental health condition resulting in disability at work are increasing due to an array of health, social and environmental factors [1, 2]. The decision whether to disclose that disability to others in the workplace and to whom, what and when to share is complex. Many chronic conditions are associated with episodic disability and include times of relative wellness punctuated by intermittent periods of activity limitations [3, 4]. Moreover, signs and symptoms of a condition may be largely invisible to others with periods of disability at work being unpredictable, even when well managed with treatment [4]. This can create disruptions in work productivity, workplace planning, and relationships. Examples of episodic or invisible disabilities include common mental health disorders like depression and anxiety, rheumatic diseases, Crohn’s and colitis, multiple sclerosis, and migraine.

Research finds that factors associated with disclosure decisions include needing to comply with workplace policies and legislation, as well as personal preferences for sharing information, need for accommodations, the extent to which the workplace is perceived as supportive, previous experiences, whether others have noticed performance problems, the desire to build trust in workplace relationships, and having to manage health crises [3, 5–20]. To date, much of the research on communication has focused on the disclosure of “stigmatized identities” [21] related to mental illness and HIV/AIDS, although some research has examined disclosure of physical conditions [9, 14, 22].

### Workplace Disclosure Goals and Outcomes

Studies often highlight that disclosure decisions are based on anticipated negative outcomes like concerns about stigma, social rejection, and potential job discrimination [13, 19, 23–27]. Fewer studies have documented anticipated positive outcomes like the receipt of instrumental and emotional support at work [5, 6, 9, 14]. Moreover, there is little research examining the reasons individuals give for disclosure or non-disclosure of health information in the workplace and the consequences of those decisions in terms of reported workplace outcomes. This may be because of the wide range of reasons and potential outcomes documented. Diverse goals and outcomes make it difficult to systematically understand disclosure-support processes, as well as the role of other variables in this process. This includes whether there are similarities or differences in disclosure related to the types of health conditions experienced, the role of work context factors, and job performance.

One theory, the disclosure processes model (DPM) [7], hypothesizes about the conditions under which disclosure goals might be linked to outcomes that are perceived as positive or negative. The DPM posits that dual goals—approach and avoidance—govern many communication decisions. Approach goals are those where individuals attempt to pursue rewarding or desired end states such as improved relationships with others or support for activities to sustain performance. Avoidance goals are attempts to avoid punishments or undesired end states such as disclosing a health condition to a supervisor to avoid being fired for poor performance. Chaudoir and Fisher note that these two types of antecedent goals can impact subsequent events because they introduce new information to others, may alter how others perceive the individual sharing information, influence the support received, or change the dynamics of future interactions in positive or negative ways [7, 28].

The DPM provides a way to conceptualize the reasons individuals give for their communication decisions and a framework for examining their relationship to outcomes. The application to a real-world situation—workers living with mental or physical health conditions who must make decisions about sharing health information at their job—is critical to better understand communication-decision processes. From a theoretical perspective, approach and avoidance goals typically are discussed as a choice between two pathways. Yet, we hypothesize that many individuals hold both types of goals for a single decision. For example, a worker may decide to disclose information about a chronic condition’s impact at work because they want to receive support for making changes to their job (an approach goal) and because they want to explain that any performance problems they’ve experienced were due to a health issue and not a lack of skills or motivation (an avoidance goal). Currently, it is not clear to what extent workers hold multiple goals and how these relate to different types of outcomes.

Also important is understanding decisions related to non-disclosure. Previous research provides numerous examples of avoidance reasons for non-disclosure such as concerns about lost opportunities at work, stigma or discrimination, and negative past experiences [5, 11, 13–15, 20, 21]. However, the DPM focuses largely on disclosure and less on decisions not to share personal information and its association with outcomes. As a result, it is not clear what kinds of reasons constitute approach goals for non-disclosure. We expect that some workers choose not to disclose, less to avoid a negative outcome, but more to maintain a positive status quo at work. For example, if an individual lives with a health condition that does not currently affect their job, they may perceive little need to disclose. This would fit with previous research finding that people disclose primarily when they believe that disclosure is effective or necessary [3, 29, 30].

The present study examined the communication experiences of an employed sample of workers living with a physical and/or mental health condition that could result in a disability at work at least some of the time. Using the DPM as a guide, we hypothesized that workers would vary in their decision whether to disclose personal health information and that their reasons could be categorized as approach or avoidance. We expected that having a greater number of approach reasons would be associated with greater perceived positive outcomes at work regardless of whether or not individuals disclose health information at work. Similarly, a greater number of avoidance reasons was expected to be associated with a greater number of negative outcomes. As noted in the DPM, individuals who pursue approach goals may be more likely to attend to the presence of positive stimuli that may signal a positive outcome or may alter interactions such that they contribute to the likelihood of a positive outcome [28, 31, 32]. In contrast, those who pursue avoidance goals may recognize the likelihood of potential negative responses from others, or their assumptions may shape subsequent interactions and communication processes that contribute to perceived negative outcomes [28, 31, 32].

### Other Factors Related to Disclosure Decisions and Outcomes

The incorporation of diverse types of information into decision making suggests that factors other than approach or avoidance goals also will play a role in understanding positive and negative workplace outcomes. For example, studies find that individuals with mental health conditions sometimes experience stigma from others at work [19, 24, 33]. As such, we expected that workers would be less likely to disclose mental health information in the workplace than physical health information. At the same time, legislation in many countries protects workers with disabilities from discrimination, including workers with mental health conditions. Mental health awareness campaigns have shed greater light on the stigma experienced by individuals and offer education and skills building for workplaces [34]. This could mean that workers who disclose their mental health condition have comparable outcomes to workers with physical health conditions. Yet, the situation may differ if health information is not communicated. Some research suggests that workers with mental health conditions who do not disclose any information about the impact of their condition on work risk being perceived by others as malingering, lacking skills or as unable to get along well with others [3]. This may be aggravated in cases of mental illness when some workers are not aware that they are in the early stages of a mental health episode [3].

Work context factors also are likely to be relevant to job outcomes in the communication process [9, 12, 14]. We hypothesized that individuals who report greater workplace support and less job stress will report experiencing more positive work outcomes. Workers employed as part of contract work, which is more precarious and less likely to have workplace supports, or workers who have less job tenure (often younger workers) are expected to report more negative outcomes. Similarly, workers who report performance problems in the form of more absenteeism, or who say they needed more accommodations at work are hypothesized to report more negative outcomes [35, 36]. This is expected to be the case regardless of whether a person has disclosed or not disclosed a disability at work.

Finally, it is not clear whether demographic variables like gender, age or education will be associated with disclosure decisions or positive and negative work outcomes. Women have a greater prevalence of some chronic conditions [37]. However, we lack data examining gender differences in workplace communication of health information. Similarly, it’s not clear whether there are age differences in disclosure of health information or whether job tenure is more relevant than the age of a worker, and what role, if any, education plays in disclosure or reports of positive and negative outcomes.

## Methods

### Study Design and Participants

Data were collected using an online, cross-sectional survey. To be eligible, participants had to be $\ge$ 18 years of age, employed for at least 15 h per week, and fluent in English. We aimed for similar numbers of participants in three age groups, 18–35 years; 36–50 years; and > 50 years. We also aimed to recruit comparable numbers of participants living with and without a physical or mental health/cognitive disability to compare their employment experiences. Participants were recruited from an existing national panel of over 1,000,000 Canadians maintained by a survey research firm and developed to be nationally representative by region and socioeconomic status. Using demographic information held by the research firm, a purposive sampling strategy was developed to identify panel members who met study eligibility criteria. Potential participants were contacted by the survey research firm and completed a short screening questionnaire to verify their eligibility. Informed consent was obtained from respondents. Among individuals meeting study eligibility and agreeing to participate, 88% completed the questionnaire. This study includes only those participants living with a disability that impacted their job at least some of the time (49.8% of the total sample). Questionnaires took 25–30 min to complete and were administered in October 2018. Ethics approval was received from the University of Toronto Research Ethics Board [REB#36184].

### Measures

#### Disability

Five questions asked participants about difficulties at their job related to: seeing or hearing; walking, using stairs, hands, fingers or other physical activities; learning, remembering or concentrating; emotional, psychological or mental health conditions; and other health problems or long-term conditions that have lasted or are expected to last six months or more. Participants could indicate that they experienced a job limitation for more than one reason (e.g., a physical and mental health limitation at work). Questions were adapted from the short disability screening questionnaire (DSQ) designed by Statistics Canada for population health surveys [38]. Items were modified to ask about difficulties with employment. Responses were on a four-point scale from 1 = no; 2 = sometimes; 3 = often; 4 = always.

#### Disclosure

Participants were asked whether they had talked to their immediate supervisor/manager about any limitations they had that might affect their work or that were related to their health or disability. Responses were Yes/No.

#### Disclosure and Non-disclosure Reasons

Based on responses to the item asking about whether participants had disclosed any limitations at work related to their health or disability, respondents saw one of two lists (disclosure reasons or non-disclosure reasons). Each list provided 10 potential reasons why an individual might have disclosed or not disclosed health information to their employer. The items drew on findings from previous studies [13, 19, 20, 23–27]. Respondents were asked whether each reason was a factor in their deciding to share information (Yes/No). Using the DPM, we divided reasons into four approach reasons for disclosure (e.g., “It’s just part of who I am”), six avoidance reasons for disclosure (e.g., “I had to explain work absences”), two approach reasons for non-disclosure (e.g., “My health or disability doesn’t affect my ability to do my job”) and eight avoidance reasons for non-disclosure (e.g., “I was concerned about lost opportunities for a promotion or new job tasks”). Scores for approach and avoidance reasons were summed separately for disclosure and non-disclosure.

#### Perceived Outcomes of Disclosing and not Disclosing Health Information

Drawing on previous research, potential positive and negative outcomes related to disclosing and not-disclosing health information were created [13, 19, 20, 23–27]. Thirteen items were developed asking participants about their perceptions of outcomes related to disclosing health information. A similar group of thirteen items asked participants about perceived outcomes related to not disclosing health information. Items included both personal and interpersonal impacts (e.g., “I spend extra effort making sure people know I’m as good as others,” “I am more stressed at work,” “people see me more positively”). Responses were on a five-point, Likert-type scale from 1 = strongly disagree; 2 = disagree; 3 = neither agree nor disagree; 4 = agree; 5 = strongly agree. Items were factor analyzed to determine scoring.

#### Job Sector

Participants indicated from a list of 21 job sectors the industry where they were employed. Responses were collapsed into four broad groups: (i) financial, insurance, business, technology, government; (ii) education, health, sciences, arts, professional; (iii) sales, service, retail; and (iv) construction, utilities, agriculture, manufacturing.

#### Contract Work

Participants were asked if they had a permanent position with their employer or were on a contract.

#### Hours Worked per Week

An open-ended question asked respondents to report the number of hours on average they worked per week. Participants working 35 or more hours per week were considered full-time employees.

#### Job Tenure

Participants were asked for the number of years and months they had worked for their current employer. Responses were rounded to the nearest year.

#### Changed Jobs

Respondents were asked whether they had changed their job or the type of work they performed as a result of their health or a disability. Responses were Yes/No.

#### Perceived Work Stress

A single item asked respondents about stress at work in the past three months. Responses were on a five-point Likert-type scale with 1 = not at all stressful; 2 = not very stressful; 3 = a bit stressful; 4 = quite a bit stressful; 5 = extremely stressful.

#### Workplace Support

Participants were asked, “to what extent do you feel that your organization is supportive of your personal needs?” Responses were on a five-point Likert-type scale with 1 = not at all; 2 = a little; 3 = somewhat; 4 = quite a bit; 5 = a great deal.

#### Accommodations Needed

Participants were asked about their need for 12 benefits, policies, or accommodations to help manage any disability at work. They included: prescription drug coverage, extended health benefits, employee assistance programs, flexibility in work schedules, modified job duties, work from home arrangements, an accessible workplace, workstation adaptations, assistive devices or technology, facilities at work to manage health, informal modifications of work, communication adaptations, other accommodations. A score was created by summing the number of accommodations respondents reported needing.

#### Days Absent

Participants were asked for the number of days they had been absent from work related to their health or a disability in the past three months, including time off because of appointments.

#### Leave of Absence

Respondents were asked whether they had taken a leave of absence (e.g., short-term disability, long-term disability, leave of absence) in the past two years because of their health or a disability. Responses were Yes/No.

#### Demographics

Information on age and education was collected. Participants were asked which category best described their gender identity: male, female, or “I do not identify as either of the above, I identify as [open-ended]”. Participants in the latter group were categorized as non-binary. We label this variable as sex/gender to account for biological and social dimensions captured by the categories.

### Statistical Analyses

Means, SDs and percentages described the disability groups, disclosure and non-disclosure reasons, perceived positive and negative outcomes, workplace characteristics and demographic variables. Continuous outcomes were checked for normality using skewness and kurtosis. Multicollinearity was checked using the variance inflation factor [39]. Disability type was categorized as physical, mental/cognitive, or both physical and mental/cognitive. Separate maximum likelihood exploratory factor analyses (EFA) were conducted with an oblique rotation and using squared multiple correlations as prior communality estimates for the 13 new items created to assess perceived outcomes of disclosure and the 13 items assessing perceived outcomes of not disclosing a health disability at work. Variance explained by each factor, root mean square residuals, the scree plot, a minimum of three items loading on each factor, and interpretability of the factors were used to select the number of factors to retain [40]. Items that had a factor loading of less than 0.4 on all factors or that loaded on more than one factor with a loading of > 0.4 were dropped from the analyses and the EFA re-run with the reduced items. This decision resulted in the loss of only one item assessing a perceived outcome related to disclosing. Based on our criteria, a two-factor solution was retained as optimum (see results for details). Cronbach’s alpha assessed the internal consistency of the factors.

Ordinary least-squared regression analyses examined the association of approach and avoidance reasons, demographic, disability type, and work context with: (1) perceived positive outcomes for disclosure of health information; (2) perceived negative outcomes for disclosure; (3) perceived positive outcomes for non-disclosure of health information; and (4) perceived negative outcomes for non-disclosure. Reference groups for gender, age, education, and disability type were men, 18–35 years old, high school or less, and physical disability. Percentages of missing data in the questionnaire were low and ranged from 0 to 5.4% item (the latter for job tenure). Data were analyzed using the Statistical Analyses System (SAS) software [41].

## Results

There were 896 participants who reported living with a chronic health condition that caused difficulty with daily activities regularly or on some occasions. Table 1 presents sample characteristics comparing participants who had disclosed their health condition to their supervisor (n = 459) with those who had not told their supervisor about their health condition (n = 437). Participants who had disclosed were similar in terms of gender, education, job sector, hours worked per week (full-time/part-time) and the mean number of needed accommodations compared to those who had not disclosed. Nearly 60% of the sample were women, about half had a post secondary education, and participants worked on average 37 h per week. Participants worked in a range of job sectors. Participants who reported disclosing their health condition at work were significantly more likely to be older, report living with both a physical and mental health condition, had greater job tenure, reported greater job stress, greater workplace support, more absenteeism, were more likely to have changed jobs in the past year and were more likely to have had a leave of absence in the past two years. They were significantly less likely to be employed in contract work (see Table 1).

**Table 1:** Sample characteristics (n = 896)

Characteristics	Disclosed	Did not disclose	P-value*
	n = 459	n = 437	
	n (%); Mean (SD)	n (%); Mean (SD)	
Sex/gender (women)	262 (57.2)	255 (58.4)	0.73
Age (years)			
18–33	133 (29.0)	164 (37.5)	0.01
36–50	156 (34.0)	144 (33.0)	
51 +	170 (37.0)	129 (29.5)	
Education			
High school or less	94 (20.5)	98 (22.5)	0.78
Some post secondary	140 (30.6)	130 (29.8)	
Post secondary	224 (48.9)	208 (47.7)	
Disability type			
Physical	102 (22.4)	114 (26.2)	0.001
Mental/cognitive	68 (15.0)	103 (23.7)	
Both physical and mental/cognitive	285 (62.6)	218 (50.1)	
Job sector			
Financial; insurance; business; technology; government	111 (24.3)	88 (20.3)	0.43
Education; health; sciences; arts; professional	147 (32.2)	149 (34.3)	
Sales; services; retail	102 (22.3)	93 (21.4)	
Construction; utilities; agriculture Manufacturing	97 (21.2)	104 (24.0)	
Contract work	31 (6.8)	53 (12.2)	0.01
Hours per week	37.6 (9.3)	37.5 (8.6)	0.92
Full-time (≥ 35 h/week)	354 (77.1)	347 (79.4)	0.40
Job tenure (years)	10.0 (9.0)	8.7 (8.8)	0.04
Changed job in past year related to health/disability	87 (19.0)	37 (8.6)	0.0001
Perceived work stress (1–5)	3.2 (1.0)	3.0 (1.0)	0.04
Workplace support (1–5)	3.2 (1.1)	2.9 (1.2)	0.001
Accommodations needed (0–12)	7.0 (3.7)	5.6 (4.1)	0.001
Days absent in past 3 months related to health/disability	5.0 (5.5)	2.6 (3.9)	0.001
Leave of absence in past 2 years related to health/disability	198 (43.1)	77 (17.7)	.0001

^*^T-test for continuous variables and chi-squared test for categorical variables. *n'*s can vary due to missing data

Table 2 presents approach and avoidance reasons for the decision to disclose or not disclose a health condition or disability at work. The percentages of respondents endorsing each reason as a factor in their decision, as well as the mean number of approach and avoidance reasons is provided. Among those who had disclosed their health at work, nearly three quarters of respondents believed their job was secure and felt it was safe to discuss (71.4%). Over 60% felt it was not a big deal, it was just part of their identity (63.4%). Over a third of respondents reported that they wanted to make changes to their job and get support (36.6%) or that others in their organization had discussed health issues and the response at work had been positive (36.3%). The most common avoidance reasons for disclosure centred on performance issues. Over 60% of respondents were concerned that their health could affect their job, so they let others know (61.0%) and nearly half of participants reported that others in their workplace had noticed changes and asked if there was a problem (47.1%). Participants also reported that their disability was getting worse and they needed to disclose (41.9%) or that they had to explain work absences (33.0%). Respondents who had disclosed their health condition at work reported on average 2.1 approach reasons for disclosure and 2.3 avoidance reasons for disclosure, *t* (452) = − 3.24, *p* < 0.001.

**Table 2:** Approach and avoidance reasons for disclosing and not disclosing a health condition or disability

Reasons for Disclosing (n = 459)	n (%); Mean (SD)
Approach reasons	
Felt job was secure and it was safe to discuss	324 (71.4)
Not a big deal; just part of who I am	286 (63.4)
Wanted to make changes to my job and get support	167 (36.6)
Others had discussed and the response was positive	165 (36.3)
Mean number of approach reasons	2.1 (1.2)
Avoidance reasons	
Health/disability could affect my job, so I let others know	278 (61.0)
Others noticed and asked if there was a problem	214 (47.1)
Health/disability was getting worse and I needed to disclose	190 (41.9)
Had to explain work absences	151 (33.0)
Started a new job and I thought others should know	118 (26.0)
Confided in someone who told others	101 (22.2)
Mean number of avoidance reasons	2.3 (1.5)

Reasons for not disclosing (n = 437)	n (%); Mean (SD)
Approach reasons	
Can manage at work without others knowing	326 (75.6)
Health/disability doesn’t affect my ability to do my job	241 (55.9)
Mean number of approach reasons	1.3 (0.8)
Avoidance reasons	
People don’t have the right to know	226 (63.3)
Nothing can be done so there’s no point in discussing	222 (51.9)
Concerned about lost opportunities for a promotion or new job tasks	174 (40.6)
Didn’t feel secure in my job	159 (37.0)
Concerned I would lose my job	158 (36.9)
The response to others with health conditions/disabilities has not been positive	138 (32.2)
Starting a new job and I didn’t want others to know	129 (30.1)
Past experiences make me concerned about sharing	113 (26.5)
Mean number of avoidance reasons	3.1 (2.5)

Total n = 896; *n*s can vary due to missing data

Among participants who had not disclosed their condition at work, 75.6% reported that they were able to manage their condition at work without needing to let others know about it and 55.9% felt their disability didn’t affect their job so there was no reason to disclose (see Table 2). Avoidance reasons for non-disclosure included that others did not have the right to the information (63.3%), nothing could be done so there was no point in letting others know (51.9%) and being concerned about lost opportunities for a promotion or new job tasks (40.6%). Over a third of respondents also reported that they did not feel secure in their jobs and did not want to disclose (37.0%) or were concerned they might lose their job (36.9%). Participants who did not disclose their health at work reported on average 1.3 approach reasons for non-disclosure and 3.1 avoidance reasons for non-disclosure, *t* (425) = − 14.16, *p* < 0.001 (see Table 2).

Separate exploratory factor analyses were conducted with the 13 items assessing outcomes related to disclosure and the 13 items assessing outcomes related to not disclosing a disability. In each case, a two-factor solution representing positive and negative outcomes for disclosure and positive and negative outcomes for not disclosing was the optimum solution. Supplemental Table 1 presents the factor loadings for the different factors.

Table 3 presents the items, mean scores and Cronbach’s alpha, a measure of internal consistency, for the perceived positive and negative outcomes of disclosure decisions. There was good variability in response to items. Cronbach’s alphas were excellent for the factors assessing positive and negative outcomes of disclosure (alpha = 0.87 and 0.90, respectively). Scores were adequate for positive outcomes of not disclosing (alpha = 0.66) and excellent for negative outcomes of not disclosing (alpha = 0.88) [42]. Among respondents who had disclosed their health or a disability, there was significantly higher agreement with statements assessing perceived positive outcomes and lower agreement with statements assessing perceived negative outcomes, *t* (454) = 9.35, *p* < 0.001. Among respondents who reported not disclosing their health condition or a disability, findings were similar. Agreement was significantly higher for statements reflecting perceived positive outcomes than perceived negative outcomes, *t* (454) = 12.26, *p* < 0.001.

**Table 3:** Perceived positive and negative outcomes associated with disclosing versus not disclosing information about a health condition or disability

Perceived outcomes	Mean (SD) score
Positive outcomes of disclosing (n = 459)	
Greater understanding of my personal needs	3.5 (1.0)
Don’t need to hide who I really am from others at work	3.5 (1.1)
Increased trust that others are looking out for my needs	3.4 (1.1)
More support at work	3.4 (1.0)
Less stress at work	3.0 (1.1)
Total positive disclosure outcomes score (5–25)	16.8 (4.3)
Cronbach’s alpha	0.86
Negative outcomes of disclosing	
Have to spend more effort to prove I’m as good as others	3.2 (1.2)
Always wondering whether others believe I’m doing a good job	3.0 (1.3)
Others view me less positively	2.6 (1.2)
Lost opportunity for promotion or new job tasks	2.6 (1.3)
Others focus on my difficulties and not my skills and abilities	2.6 (1.2)
Others gossip about my personal situation at work	2.6 (1.3)
Experienced rejection or stigma from others	2.5 (1.2)
Total negative disclosure outcomes score (7–35)	19.0 (6.9)
Cronbach’s alpha	0.90

Perceived outcomes	Mean (SD); Total score
Positive outcomes of not disclosing (437)	
My job duties remain the same	3.9 (0.9)
People see me more positively	3.6 (0.9)
People focus on my skills and abilities	3.5 (1.0)
I don’t wonder whether others believe I’m doing a good job	3.4 (1.0)
Others don’t gossip about my personal situation	3.3 (1.1)
Total positive non-disclosure outcomes (5–25)	17.8 (3.1)
Cronbach’s alpha	0.66
Negative outcomes of not disclosing	
People don’t understand my personal needs	3.2 (1.0)
I am more stressed	3.2 (1.1)
I have to work harder to make sure people know I’m as good as others at my job	3.1 (1.1)
I have less trust that others are looking out for my needs	3.1 (1.0)
I have to hide who I really am from others	3.0 (1.2)
I have less support at work	2.9 (1.1)
I have experienced rejection or stigma from others	2.8 (1.2)
I have lost opportunities for promotion or new job tasks	2.5 (1.2)
Total negative non-disclosure outcomes (8–40)	23.8 (6.8)
Cronbach’s alpha	0.88

Total n = 896; n’s can vary due to missing data

We conducted bivariate analyses examining the factors associated with positive and negative outcomes of disclosure and non-disclosure of a health condition or disability at work. We examined demographic factors (age, gender, education); type of disability; work context variables (job sector, contract work, hours worked per week, job tenure); work perceptions (e.g., work stress, workplace support, accommodations needed) and absenteeism. Variables significant at a bivariate level of p < 0.05 for at least one of the four outcomes were included in all multivariable analyses. This ensured that the same variables were included across analyses.

Table 4 presents the results of the multivariable analyses for perceived positive and negative outcomes of disclosure of a health condition or disability at work. Participants reporting a more supportive workplace and who had a greater number of approach reasons for disclosure reported more positive outcomes of disclosure. Women were significantly less likely to report positive outcomes than men. Participants reporting greater work stress also were significantly less likely to report positive outcomes. Avoidance reasons for disclosure, work stress, needing more accommodations, and a greater number of days absent were significantly associated with negative outcomes among those who disclosed their health at work. Greater workplace support and more approach reasons for disclosure were significantly related to less negative outcomes of disclosure.

**Table 4:** Multivariable analyses of factors associated with perceived positive and negative outcomes in disclosing information of a health condition or disability

Factors	Perceived positive outcomes (n = 441)	Perceived negative outcomes (n = 442)
	Beta (95% CI)	Beta (95% CI)
Gender (women)	− 0.69 (− 1.37, − 0.02)*	− 0.99 (− 2.13, 0.16)
Age (years)		
18–35	–	–
36–50	− 0.10 (− 0.95, 0.75)	− 0.33 (− 1.76, 1.11)
51 +	0.19 (− 0.68, 1.07)	− 0.85 (− 2.32, 0.63)
Education		
High school or less	–	–
Some post secondary	70.30 (− 0.64, 1.24)	0.69 (− 0.89, 2.26)
Postsecondary	− 0.38 (− 1.24, 0.49)	0.87 (− 0.59, 2.33)
Disability type		
Physical	–	–
Mental/cognitive	0.28 (− 0.88, 1.43)	0.94 (− 1.02, 2.89)
Both physical and mental/cognitive	− 0.17 (− 1.02, 0.69)	0.74 (− 0.70, 2.19)
Work stress	− 0.47 (− 0.85, − 0.10)*	1.16 (0.53, 1.79)**
Workplace support	1.36 (1.05, 1.67)**	− 1.38 (− 1.91, − 0.85)**
Accommodations needed	0.02 (− 0.07, 0.12)	0.37 (0.21, 0.53)**
Days absent	− 0.02 (− 0.08, 0.05)	0.14 (0.03, 0.25)*
Approach reasons for disclosure	1.03 (0.71, 1.34)**	− 0.99 (− 1.52, − 0.47)**
Avoidance reasons for disclosure	0.05 (− 0.21, 0.31)	0.74 (0.30, 1.18)**

^*^p < .05; **p < .01

Table 5 presents the multivariable analyses of variables associated with positive and negative outcomes of not disclosing a health condition or disability at work. The only variables associated with perceived positive outcomes of not disclosing were a greater number of approach and avoidance reasons for not disclosing. However, a range of variables were associated with perceived negative outcomes of not disclosing a health condition or disability at work. Having mental health or both physical and mental health disability, greater work stress, needing more accommodations and having more avoidance reasons were associated with more perceived negative outcomes related to the decision not to disclose. Post-secondary education, more workplace support and more approach reasons for not disclosing were associated with fewer perceived negative outcomes.

**Table 5:** Multivariable analyses of factors associated with perceived positive and negative outcomes in not disclosing information of a health condition or disability

Factors	Perceived positive	Perceived negative
	Outcomes (n = 419)	Outcomes (n = 420)
	Beta (95% CI)	Beta (95% CI)
Gender (women)	0.43 (− 0.18, 1.03)	− 0.27 (− 1.3, 0.76)
Age (years)		
18–35	–	–
36–50	0.12 (− 0.60, 0.84)	− 0.44 (− 1.66, 0.79)
51 +	0.04 (− 0.76, 0.84)	0.19 (− 1.16, 1.55)
Education		
High school or less	–	–
Some post secondary	0.40 (− 0.42, 1.23)	− 1.42 (− 2.82, − 0.02)*
Postsecondary	0.41 (− 0.35, 1.17)	− 0.62 (− 1.91, 0.68)
Disability type		
Physical	–	–
Mental/cognitive	− 0.17 (− 1.07, 0.72)	1.67 (0.15, 3.19)*
Both physical and mental/cognitive	− 0.66 (− 1.43, 0.11)	1.45 (0.14, 2.75)*
Work stress	− 0.24 (− 0.58, 0.11)	0.58 (− 0.00, 1.17)*
Workplace support	0.14 (− 0.14, 0.42)	− 1.46 (− 1.93, − 0.98)**
Accommodations needed	− 0.01 (− 0.09, 0.07)	0.19 (0.06, 0.32)**
Days absent	0.04 (− 0.04, 0.12)	0.12 (− 0.02, 0.26)
Approach reasons for non-disclosure	0.78 (0.36, 1.21)**	− 1.23 (− 1.95, − 0.51)**
Avoidance reasons for non-disclosure	0.18 (0.04, 0.32)**	1.09 (0.85, 1.32)**

^*^p < .05; **p < .01

## Discussion

Living with a disability can create challenges in sustaining employment. This study illuminates the complex goals that workers with a disabling health condition hold when deciding whether to share personal health information at work, and their perceptions of the outcomes of their decision. We used a theoretical model, the disclosure processes model (DPM), to categorize the diverse approach and avoidance reasons participants reported as important to their decisions. Our research is novel in including individuals with physical and mental health disabilities and in its focus not only on perceived negative outcomes but also positive outcomes of disclosure decisions. The findings highlight that, as expected, approach and avoidance reasons were significant factors associated with perceived positive and negative outcomes of disclosing/not disclosing along with work context and other factors. Also as hypothesized, most participants had multiple approach and avoidance reasons for their decisions and often reported positive outcomes, regardless of whether they had disclosed or not disclosed information about a disability at work. At the same time, many participants indicated agreement with a variety of negative outcomes of their decisions. These findings highlight the need for greater organizational attention to communication-support processes, including understanding a worker’s disclosure goals, to better sustain the employment of individuals working with a physical or mental health disability.

### Disclosure Decisions and Approach-Avoidance Reasons

There was variability in whether participants with a disabling health condition had disclosed information to their supervisor with about half the sample reporting disclosing and half not disclosing. Like previous research, those who disclosed appeared to have a greater need for support [5, 9, 14, 24]. They reported more absenteeism, were more likely to live with both a physical and mental health condition, reported more work-related stress, were more likely to have changed jobs because of their disability and have a leave of absence in the previous two years. Similar to other research, they also reported a better psychosocial work environment, including greater workplace support and having worked for their employer for a longer tenure [5, 9, 14, 24]. The reasons participants endorsed for their disclosure provided additional insight into issues of need and support. There was variability in the reasons endorsed by participants with no single approach or avoidance reason appearing to drive decision making. Most common were perceptions of the workplace as supportive and personal acceptance of living with a disability as a part of one’s identity. In addition, being proactive and monitoring one’s health to inform others was commonly reported. Less common were reasons where participants felt forced to disclose because of a negative impact on their performance. These findings differ from studies reporting the perspectives of organizational representatives like supervisors, human resources professionals, union representatives and labour lawyers. In the latter research, absenteeism and performance often were raised by organizational representatives as precipitating disclosure discussions [3, 33]. This discrepancy may be because organizational representatives often do not learn about a disability when it is well managed by the worker or when performance impacts are avoided with proactive instances of support or readily available accommodations like flextime [36]. Additional research is needed to better understand proactive versus reactive reasons for disclosure and to better document successes and not just challenges in disability-support processes.

When respondents did not disclose a disability at work, they again typically held multiple reasons for their decision. Approach reasons for non-disclosure requires greater attention in future studies. We characterized the absence of need, either because a respondent was able to manage their disability or because their health did not impact their work, as an approach reason because individuals wanted to maintain a positive status quo. These reasons were among the most frequently endorsed by participants. Additional qualitative and quantitative research is needed to expand on these and other approach reasons for non-disclosure to provide a better balance of approach and avoidance reasons and to improve the measurement of approach goals for not disclosing a disability at work. Research is also needed with additional samples of employed and not employed individuals to look at the extent to which individuals living with a disability believe they can successfully manage their disability at work. There may be a healthy worker effect that explains some of our findings with those whose conditions are well managed or less severe being more likely to be employed and those with worse health being less likely to be able to work.

Concerns about stigma and negative workplace repercussions have been highlighted previously as guiding non-disclosure decisions [13, 19, 23–27]. In this study, avoidance goals like worries about losing a job or opportunities for promotion and past negative experiences were reported by fewer respondents than approach goals. This may be because many participants had a relatively long work history with their current employer, and most were not in contract work. Despite this, concerns were expressed by a third to 40% of participants, despite legislation protecting individuals working with a disability. This indicates that workplaces need to devote greater attention to fostering positive work environments, addressing implicit biases that shape attitudes and behaviours toward workers living with a disability, and ensuring that workplace practices are not prejudicial or discriminatory.

### Perceived Positive and Negative Disclosure Outcomes

Respondents reported varying degrees of agreement with diverse positive and negative outcomes related to their decision to disclose or not disclose their disability at work. They expressed significantly greater agreement with positive outcomes of their decision, regardless of whether they had decided to disclose or not disclose health information. This underscores that there was no single “right” decision for a worker living with a disability with workers often believing they had made a good decision based on their own needs. At the same time, the outcomes created for this study tended to focus on personal and interpersonal outcomes. Additional research is needed to include organizational outcomes such as reduced absenteeism, improved workplace performance, and return to work after a leave of absence.

Multivariable analyses examined the association of approach and avoidance reasons with perceived outcomes of participants’ decisions. As hypothesized, approach and avoidance reasons were consistently associated with perceived positive and negative outcomes after controlling for other factors. A greater number of approach goals were associated with greater perceived positive outcomes and less negative outcomes among those who disclosed as well as among those who did not disclose health information. Avoidance goals were associated with greater perceived negative outcomes for both disclosure and non-disclosure of health information. These findings are cross-sectional and do not suggest a causal direction or that outcomes are determined by simply selecting an approach or avoidance goal. Instead, several mechanisms are potentially at play. The reasons endorsed in this study showed that participants were appraising their health and social environment as hypothesized by DPM [7]. It is perhaps not surprising that those who reported positive past experiences, a supportive work environment and that their job was secure also reported more positive outcomes of disclosure like greater understanding of personal needs and less stress at work. By the same token, those who reported they had not disclosed information about their disability because nothing could be done to help, didn’t feel secure in their job, or because they had poor past experiences were more likely to report negative outcomes. Future research, especially longitudinal studies, need to explore to a greater degree the different types of goals and reasons participants give for their decisions and their relationship to workplace outcomes and well being. Research is also needed to explore how information changes interpersonal dynamics, including from the perspectives of supervisors and co-workers who have to act on new information from a worker who discloses a disability, or who are making judgements about information in trying to explain a worker’s behaviour who has not disclosed information [3, 7, 43, 44].

As hypothesized, a greater number of approach reasons was associated with positive outcomes for non-disclosure decisions. Of interest is that a greater number of avoidance reasons also were associated with positive outcomes for non-disclosure. Looking further at the reasons our participants endorsed suggests that attempts to preserve a status quo underlie many decisions. Approach reasons included having no problems and perceiving no need to disclose. This was associated with positive outcomes like job duties remaining the same, being viewed positively by others and avoiding gossip. Avoidance reasons for not disclosing included concerns about the security of one’s job and experiencing negative past experiences. Not disclosing may ensure these outcomes do not arise and, again, preserve the current status quo. However, a greater number of avoidance reasons also were associated with negative outcomes like others not understanding one’s needs, increased stress, and having to work harder to prove oneself. A more complete understanding of the processes by which avoidance goals are associated with different outcomes is needed. Previous research finds that workers report that others sometimes view them as unmotivated or malingering if their work is affected by a chronic condition causing a disability and others are not aware of the cause [45]. This study suggests that avoidance reasons for not disclosing may act as a double-edged sword, preserving a positive status quo only as long as there do not exist other negative behavioural or performance changes.

As expected, other factors were related to positive and negative outcomes of disclosure and non-disclosure. We had expected that participants with mental health conditions would be less likely to disclose but more likely to report negative outcomes when not disclosing, possibly because of unexplained behavioural or performance indicators that signal challenges at work. Our findings showed that participants with mental health conditions were no less likely to disclose a disability than individuals with a physical disability. However, participants living with both a physical and mental health condition were more likely to disclose, possibly because their disability resulted in a greater need for support at work. As hypothesized, participants living with a mental health condition or both a physical and mental health condition were more likely to report negative outcomes when not disclosing their disability at work. The findings are interesting as previous research would suggest that the non-disclosure of a mental health condition is intended to avoid stigma. Additional research is needed, but these findings suggest that, despite not disclosing a disability, individuals may “leak” signs of a disability in their behaviours or workplace performance, which is interpreted negatively by others as motivation or skills problems or a negative interpersonal style [3, 5, 11].

Although there were no differences between women and men in the decision to disclose, women reported fewer positive outcomes of disclosure than men. There were no gender differences in negative outcomes. The reasons for this are unclear. Other research finds a number of differences in the support and accommodation experiences of women living with a disability. This includes that women have been found to report more accommodation needs and greater unmet needs [36]. It may be that one of the reasons that women report fewer positive outcomes is that they experience more challenges in receiving support. Going forward, a sex and gender-based analysis (SGBA) of disclosure decisions, workplace context, and support outcomes is needed. This research could help illuminate similarities and differences between women and men in the biological bases of physical and mental health conditions (sex differences), and in roles, perceptions, and cultural dimensions in disclosure decisions and workplace context (gender differences) that may underpin outcomes and potential workplace inequities [46–50].

As noted earlier, work context factors like job stress, a lack of workplace support, need for accommodations and absenteeism were related to being more likely to disclose a disability at work. Analyses also found these factors associated with disclosure outcomes, particularly negative outcomes. This was true in cases where the decision was to disclose or not disclose. The findings highlight areas for workplace intervention and underscore that it is often not the decision whether or not to disclose that makes the most difference—it’s the psychosocial work environment or workplace culture in which decisions are made that matters. Workers living with a disability who also contend with a stressful or less supportive workplace environment may be in a lose-lose situation no matter what their disclosure decision.

This study has several strengths and limitations. We drew on a theory to guide the development of new measures to assess approach and avoidance decisions and perceived outcomes related to disclosing a disability at work. Our sample was relatively large and diverse, but data were cross sectional. This is a limitation not only in trying to tease apart the causal direction of disclosure goals and outcomes, but also in inadvertently portraying disclosure decisions as one-time events. In fact, disclosure decisions typically are ongoing, evolving processes [7, 9]. Longitudinal research is needed to better understand the relationships between goals and outcomes, as well as changes in decisions and who is involved in receiving information. Our study is among the first to examine approach and avoidance goals and their relationship to positive and negative outcomes. We were able to categorize a wide range of participant goals and link them to different types of outcomes. At the same time, research is needed to replicate and continue this research, especially the measurement of approach and avoidance goals, as well as the measurement of outcomes. Particularly helpful would be to expand the outcomes of interest to include organizational outcomes like reduced absenteeism, improved performance and return to work. Also needed is additional information about work contexts as a determinant of approach avoidance goals and diverse outcomes. It is important to acknowledge potential sample selection biases that may be present in these data. We lack detailed information about the panel from which respondents were drawn and response rates. The study also did not include workers had given up employment because they chose not to disclose a health condition or because they were not able to receive workplace support. Finally, we are lacking information about disclosure and support changes over time, including the extent to which workers change jobs in order to find more supportive work environments. Longitudinal data and qualitative studies that more fully examine disclosure and work contexts, including the experiences of individuals who have given up employment are needed.

Despite these limitations, this study is one of the first to undertake an examination of the disclosure decision goals of workers living with a disability because of a physical and/or mental health condition and the association between these decisions and perceived outcomes. The findings highlight benefits and challenges that workers perceive arise when they choose to disclose or not disclose personal health information. By better understanding disclosure decision making, we can inform organizational health privacy and support gaps and identify new directions that can enable workers with disabilities to better sustain their employment or return to work.

## Supplementary Information

Below is the link to the electronic supplementary material.

Electronic supplementary material 1 (DOCX 34 kb)
