# Supplementary material for: Does it matter what your reasons are when deciding to disclose (or not disclose) a disability at work? The association of workers’ approach and avoidance goals with perceived positive and negative workplace outcomes
Source: J Occup Rehabil. 2021 Feb 1;31(3):638–51. doi: 10.1007/s10926-020-09956-1 (PMC8298367; doi:10.1007/s10926-020-09956-1)
Supplement: Supplementary file 1 — Electronic supplementary material 1 (DOCX 34 kb) [file 10926_2020_9956_MOESM1_ESM.docx]

Supplemental Table 1: Factor loadings for a two-factor solution of perceived positive and negative outcomes for disclosing or not disclosing a health condition or disability at work

**___________________________________________________________________________**

Perceived Outcomes Factor 1 Factor 2

___________________________________________________________________________

Positive outcomes of disclosing

Greater understanding of my personal needs **0.81** 0.04

Don’t need to hide who I really am from others at work **0.58** -0.22

Increased trust that others are looking out for my needs **0.85** 0.00

More support at work **0.86** 0.05

Less stress at work **0.67** 0.06

Cronbach’s alpha 0.86

Negative outcomes of disclosing

Have to spend more effort to prove I’m as good as others 0.12 **0.61**

Always wonder whether others believe I’m doing a good job 0.03 **0.70**

Others view me less positively -0.01 **0.79**

Lost opportunity for promotion or new job tasks 0.00 **0.78**

Others focus on my difficulties and not my skills and abilities -0.05 **0.79**

Others gossip about my personal situation at work -0.04 **0.75**

Experienced rejection or stigma from others 0.03 **0.87**

Cronbach’s alpha 0.90

Factor correlation -0.29, 95% CI (-0.38, -0.20)

Supplemental Table 1 continued: Factor loadings for a two-factor solution of perceived positive and negative outcomes for disclosing or not disclosing a health condition or disability at work

**___________________________________________________________________________**

Perceived Outcomes Factor 1 Factor 2

___________________________________________________________________________

Positive outcomes of not disclosing

My job duties remain the same **0.59** -0.11

People see me more positively **0.66** 0.06

People focus on my skills and abilities **0.53** 0.14

I don’t wonder whether others believe I’m doing a good job **0.50** 0.07

Others don’t gossip about my personal situation **0.43** -0.21

Cronbach’s alpha 0.66

Negative outcomes of not disclosing

People don’t understand my personal needs 0.09 **0.64**

I am more stressed -0.08 **0.78**

I have to work harder to make sure people know I’m as

good as others at my job 0.09 **0.70**

I have less trust that others are looking out for my needs 0.08 **0.75**

I have to hide who I really am from others -0.01 **0.76**

I have less support at work -0.08 **0.78**

I have experienced rejection or stigma from others -0.03 **0.66**

I have lost opportunities for promotion or new job tasks -0.09 **0.66**

Cronbach’s alpha 0.88

Factor correlation 0.16, 95% CI (0.05, 0.26)

_____________________________________________________________________________

Total n = 896
